# Management and referral for high-risk conditions and complications during the antenatal period: knowledge, practice and attitude survey of providers in rural public healthcare in two states of India

**DOI:** 10.1186/s12978-019-0765-y

**Published:** 2019-07-10

**Authors:** Samiksha Singh, Pat Doyle, Oona M. R. Campbell, G. V. S. Murthy

**Affiliations:** 1 Indian Institute of Public Health-Hyderabad, Public Health Foundation of India, Amar Co-operative society, Plot#1 AVN Arcade, Kavuri Hills, Madhapur, Hyderabad, Telanagana 201010 India; 2 Department of Non-communicable disease epidemiology, Faculty of Epidemiology and Population Health, London School of Hygiene and Tropical Medicine, London, UK; 3 Department of Infectious Disease Epidemiology, Faculty of Epidemiology and Population Health, London School of Hygiene and Tropical Medicine, London, UK; 4 Department of clinical research, Faculty of Infectious and Tropical Diseases, London School of Hygiene and Tropical Medicine, London, UK

**Keywords:** Pregnant women, Pregnancy, Obstetric, Obstetric high-risk, Obstetric complication, Maternity services, Quality

## Abstract

**Background:**

Appropriate antenatal care improves pregnancy outcomes. Routine antenatal care is provided at primary care facilities in rural India and women at-risk of poor outcomes are referred to advanced centres in cities. The primary care facilities include Sub-health centres, Primary health centres, and Community health centres, in ascending order of level of obstetric care provided. The latter two should provide basic and comprehensive obstetric care, respectively, but they provide only partial services. In such scenario, the management and referrals during pregnancy are less understood. This study assessed rural providers’ perspectives on management and referrals of antenatal women with high obstetric risk, or with complications.

**Methods:**

We surveyed 147 health care providers in primary level public health care from poor and better performing districts from two states. We assessed their knowledge, attitudes and practices regarding obstetric care, referral decisions and pre-referral treatments provided for commonly occurring obstetric high-risk conditions and complications.

**Results:**

Staff had sub-optimal knowledge of, and practices for, screening common high-risk conditions and assessing complications in pregnancy. Only 31% (47/147) mentioned screening for at least 10 of the 16 common high-risk conditions and early complications of pregnancy. Only 35% (17/49) of the staff at Primary health centres, and 51% (18/35) at Community health centres, mentioned that they managed these conditions and, the remaining staff referred most of such cases early in pregnancy. The staff mentioned inability to manage childbirth of women with high-risk conditions and complications. Thus in absence of efficient referral systems and communication, it was better for these women to receive antenatal care at the advanced centres (often far) where they should deliver. There were large gaps in knowledge of emergency treatment for obstetric complications in pregnancy and pre-referral first-aid. Staff generally were low on confidence and did not have adequate resources. Nurses had limited roles in decision making. Staff desired skill building, mentoring, moral support, and motivation from senior officers.

**Conclusion:**

The Indian health system should improve the provision of obstetric care by standardising services at each level of health care and increasing the focus on emergency treatment for complications, appropriate decision-making for referral, and improving referral communication and staff support.

## Plain English summary

Appropriate care during pregnancy improves outcomes of childbirth. The advanced centres in cities provide specialist care. While the primary care in rural India has 3 levels—first Sub-centres, second the Primary health centres, and third the Community health centres—that should provide basic and comprehensive care. We surveyed health care providers at the primary levels, to assess their perspectives on obstetric care—screening, referral decisions and treatments—provided for commonly occurring high-risk conditions and complications of pregnancy. The study centres provided fewer services than that expected at respective level. Staff had sub-optimal knowledge of, and practices for, screening common high-risk conditions and assessing complications in pregnancy. If detected any, all staff from the Sub-centre referred such cases without any management. A quarter of the staff from Primary health centres, and a half from the Community health centres managed common conditions, and the remaining staff referred most of these cases early in pregnancy. The latter two levels should be able to provide appropriate care but they were under-confident and even if they could manage they felt it better for the women with high-risk or complication to receive antenatal care at the advanced centre where she should deliver. There were large gaps in knowledge of emergency treatment for obstetric complications and pre-referral first-aid. The Indian health system should improve the provision of obstetric care by standardising services at each level of care and increasing the focus on emergency treatment for complications, appropriate decision-making for referral, and improving referral communication and staff support.

## Background

Globally, an estimated 830 mothers die from preventable causes every day, of which 99% are in the low and middle income countries (LMIC) [1]. Between 1990 and 2015, the maternal mortality reduced worldwide by 44% to 216/100,000 live births [2], and in India it reduced by 67% [2] from estimated 400 in 1990 to 130 per 100,000 live births in 2015 [3]. The Sustainable Development Goal target is to reduce this ratio to 70 by 2030 in the world [4] and India. Maternal deaths are most common among adolescents, poor women, and those from rural populations [5]. Although most of these deaths occur in the intrapartum and immediate post-partum period, evidence suggests a large proportion of maternal deaths are a consequence of the poor quality of preventive and promotive antenatal care, missed or delayed diagnosis in pregnancy, or poor management of complications in pregnancy [5, 6].

Prevention and management of high-risk conditions and early complications in pregnancy begins with the pre-conception period and lasts throughout pregnancy [7]. WHO (2015) standards for improving the quality of antenatal care for a positive pregnancy experience focus on basic preventive and promotive antenatal care and recommend early assessment for high-risk cases and complications in pregnancy [2]. These guidelines are to guide clinical decisions and are for primary level of health care, either at the facility or in the community. Another WHO guideline (revised in 2017) describes the management of obstetric complications at the district level and mentions these with respect to the period of gestation [8]. However, the use of these guidelines at different levels of facilities, and referral systems, in low resource countries is not well understood. The defined levels of emergency obstetric care (EmOC)—comprehensive EmOC (CEmOC), basic EmOC (BEmOC) and birthing centres [5]—focus primarily on childbirth, and fail to consider complication management in early pregnancy. Whilst the level of EmOC care available defines a facility’s capabilities for managing complications in early pregnancy, it is observed in several low resource settings that centres designated as EmOC may not actually be able to provide all the signal functions [9–13]. Health workers from such settings which cannot manage complications should thus be highly competent in prevention, risk assessment, stabilisation of complicated cases, and arranging transfer and care at functional higher referral levels [14, 15].

In India, the guidelines for Skilled Birth Attendants (SBA) and management of obstetric complications provide guidance for clinical management and decision making at the primary level of care. These guide in managing the low-risk as well as common high-risk conditions and complications during pregnancy, childbirth and postpartum care, and suggest referral if the facility is incapacble of managing the case [16]. We found very few studies from India elaborating on the providers’ perspective on understanding and management of high-risk conditions and complications in pregnancy, particularly in the antenatal period [17–19]. This understanding is essential for improving the quality of antenatal care, early management of complications and continuum of care through appropriate referrals. Thus, we planned our study in rural public primary care facilities in India with objectives of- 1) assessing the knowledge, attitudes and practices of primary care providers about screening and referral of high-risk conditions and complications in the antenatal period, and 2) determining the facilitators and challenges to appropriate referrals in antenatal care in a three-tier public health system.

### Levels of obstetric care in rural public health system in India

There are four levels of obstetric care in primary health care system for rural India, however, the services provided at these centres are not standardized [12, 20]. Amongst the peripheral health facilities, the lowest rung of the ladder are the Sub-health centres (SHCs), which provide antenatal care primarily, and birthing services rarely; next level is Primary health centres (PHCs) that provide either BEmOC or only birthing services. The higher level is Community health centres (CHC) that should provide CEmOC care, but mostly only provide BEmOC or even less [12, 20, 21]. Above these are the referral facilities that provide CEmOC and specialist care.

## Methods

We conducted a cross-sectional Knowledge Attitude and Practice (KAP) survey amongst staff from peripheral health centres in two purposively selected Indian states-Himachal Pradesh from the North with a hilly terrain, and Andhra Pradesh (prior to its division into two states) from the South with plains and tribal pockets. Both the states have better health indicators compared to the country’s average but have variable rural health infrastructure. (Refer to Annex-1 for details).

We obtained permissions from the state and district officials, and sought their support in sampling and connecting to the facilities. Based on the performance ranking by the health department of India [22], we randomly selected two districts from Himachal Pradesh: one poor and one good performing, and three districts from Andhra Pradesh: one poor, one average, and one good performing district. We randomly selected two CHCs known to be working as BEmOCs from each of the districts. In each CHC catchment area, we randomly selected two PHCs that were providing birthing services and, two SHCs per chosen PHC. In the two districts from Himachal Pradesh, we could not find two BEmOC CHCs, so we selected Sub-district hospital (higher level than CHC) providing BEmOC. We additionally selected three PHCs in Himachal Pradesh that only provided antenatal care as they represented a larger pool of PHCs here. We included all the doctors and staff nurses posted in labour rooms at small CHCs and PHCs. In Sub-district hospitals or big CHCs, where there were more than two staff nurses, we included the two present in the labour room on the day of our visit. These centres did not have ANMs, but had supervisors for ANMs posted in linked SHCs. There were no doctors and staff nurses for SHCs. There were no refusals to participate.

We developed tools for the KAP and the facility survey in English, and translated them into local languages. We pre-tested and validated the tools before use. Interviews were conducted mostly by one of the authors (SS), but about a third was done by a research assistant because of language barriers in Andhra Pradesh. Both the interviewers were trained public health professionals with more than 5 years research experience.

The KAP survey was conducted using a survey tool that consisted of a mix of structured questions and 2 open ended questions. The survey tool was administered by the interviewer, who asked questions but did not prompt the responses. Based on the interviewee responses he checked on the appropriate pre-listed options, and took notes for additional information. During the interviews, we assessed the knowledge, attitudes, and practices of health staff regarding screening and referral for high-risk and early complications in pregnancy. We used scenarios (vignettes) with three common obstetric ailments (moderate anaemia, pre-eclampsia and pre-term labour pains) to test the ability of staff to diagnose particular cases and to assess the treatment to be provided for stabilisation and decisions regarding referral. The un-prompted reponses were recorded against the pre-listed options. We assessed administrative processes followed for referrals. We also asked two open ended questions about the problems faced in the referral of pregnant women and about the support they needed from the health systems to improve the quality of referrals for pregnant women. There is a possibility of information bias where the actual practice may be different from that stated in the interview and also social desirability bias. To encourage honest responses, we specified clearly that the information obtained from the providers will be strictly kept confidential and will have no negative bearing on them.

To understand the context better, we conducted brief facility surveys at the chosen health centres using facility checklists based on Indian Public Health Standards and the services to be provided for the level of the health centre with respect to emergency obstetric care [23]. We interviewed the head of the obstetric team about the staff, and services provided in past 3 months, extracted information about benefeciaries and outcomes from the registers in past 6 months, and physically verified the infrastructure at the time of our visit. We found that as per State policy, SHCs were neither promoted for nor provided with the infrastructure for delivery care, so we did not do facility surveys in SHCs. We made several observations during interviews and observed a small sample of patients being provided antenatal care during our visit. There was no devised research plan for these observations, but we made use of the opportunity and made notes on general functioning in the facilities.

In this study, high-risk conditions in pregnancy comprised of demographic, obstetric or medical conditions that could elevate the risk of occurrence of a complication in pregnancy. A complication in pregnancy refers to any medical complication that developed during pregnancy labour or childbirth or within 42 days of termination of pregnancy, or is a pre-existing condition exacerbated as a consequence of pregnancy.

**Fig. 1:**
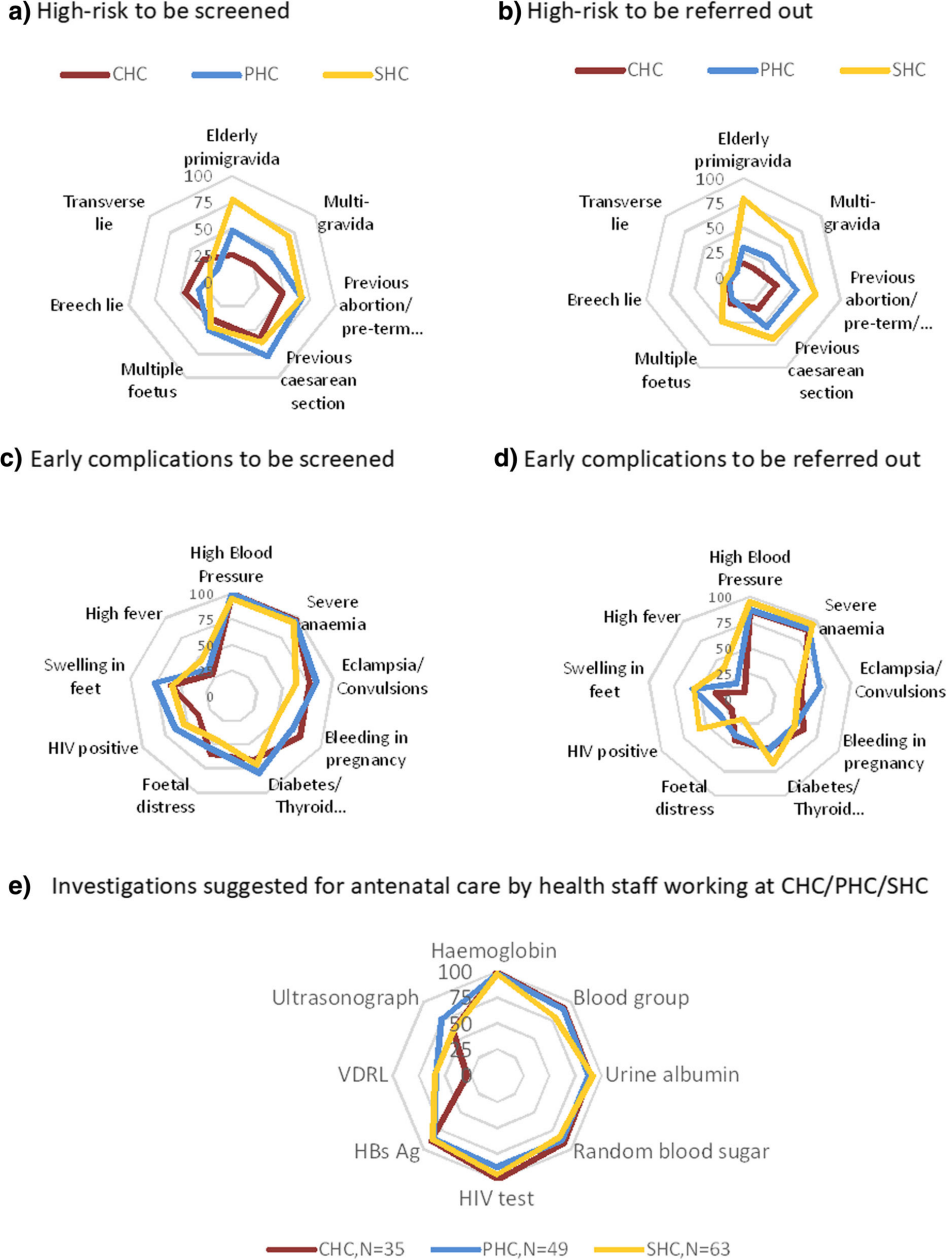
Knowledge about high-risk and early complications in antenatal period to be screened and referred out, among health staff working at CHC/PHC/SHC, %. (*In these spider diagrams the centre is 0% and the outermost circle is 100%. Overlap of lines mean small difference in the health facility. Legend shows the N for each type of facility*)

**Fig. 2:**
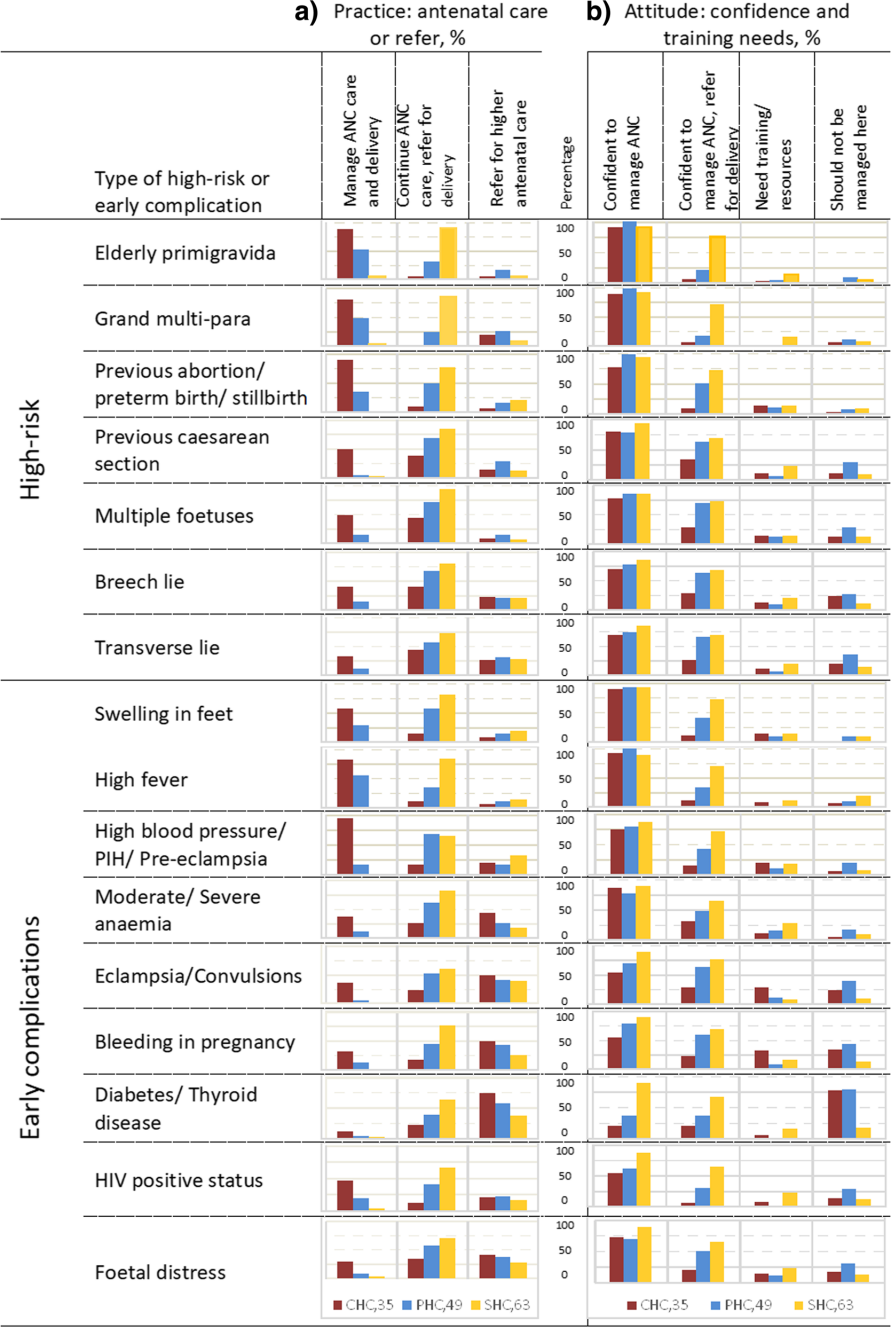
Practice and attitude regarding high-risk or early complication in pregnancy. Data presented as proportion of staff at CHC/PHC/SHC responding yes to each question. (*Legend shows the N for each type of facility. The centre column represents y axis in % for graphs in each row*)

For analysis, data were recorded on MS excel worksheets and imported to STATA 13.0 for data cleaning and analysis. We summarised the profile of respondents. For knowledge assessment on the conditions to be screened and referred, we computed proporitions of participants (N) who mentioned (unprompted) the high-risk conditions (nH1, nH2, …nH7) and complications (nC1, nC2, …nC9), each (Fig. 1). We asked about the providers’ practices and attitude for each of the listed conditions with options provided beforehand (Fig. 2). We computed proportion of responses for each condition asked. For overall knowledge, attitude, and practice, we computed the proportion of participants who mentioned appropriately for 10 of the 16 common conditions under study. The results are presented separately by level of the health centre. Information from facility checklists were described separately for Sub-district hospitals/CHCs and PHCs. We summarise the information obtained from open ended responses and other observations.

**Table 1:** Characteristics of health staff who participated in the KAP survey

	SHC *N* = 63	PHC *N* = 49	Sub-district hospital /CHC *N* = 35
Designation of participants, (%)			
*Doctor*	*0*	*18 (37%)*	*13 (37%)*
*Staff nurse*	*0*	*31 (63%)*	*22 (63%)*
*ANM*	*63 (100%)*	*0*	*0*
Mean years of experience (95% CI)	14.5 (12.3–16.7)	9.5 (7.5–11.5)	10.4 (7.9–12.9)
Mean years of service in current centre (95% CI)	7.2 (5.7–8.6)	4.3 (3.4–5.1)	5.0 (3.6–6.3)
SBA trained, (%)	31 (49%)	38 (78%)	23 (66%)
Doctor*	*–*	*11 (61%)*	*7 (54%)*
Staff nurse*	*–*	*27 (87%)*	*16 (73%)*
ANM*	*31 (49%)*	*–*	*–*
Mean years since SBA training (95% CI)	3.7 (1.8–7.7)	3.1 (2.1–4.0)	2.3 (1.6–3.1)
Trained for Safe Childbirth Checklist, (%)	9 (14%)	22 (45%)	12 (34%)
Doctor*	*–*	*8 (44%)*	*4 (31%)*
Staff nurse*	*–*	*14 (45%)*	*8 (36%)*
ANM*	*9 (14%)*	*–*	*–*
Number of deliveries assisted/ supervised in past 6 months; median (IQR)	4 (0–8)	28 (20–40)	32 (20–60)
Doctor**	*–*	*28 (3–60)*	*50 (25–80)*
*Staff nurse*	*–*	*33 (20–52)*	*30 (20–50)*
*ANM*	*4 (0–8)*	*–*	*–*

SBA = Skilled Birth Attendant; ANM = Auxiliary Nurse Midwife; * Percentage out of number of participants with type of designation; **Doctors mostly did not assist deliveries but reported the deliveries they supervised directly or over phone

**Table 2:** Services available for management of childbirth by type of health centre (*N* = 29 centres included in facility survey)

	PHC, *N* = 15	Sub-district hospital/CHC, *N* = 14
Basic birthing services, %		
Sterilised equipment	13 (87%)	14 (100%)
Injection oxytocin 10 IU within 1 min of delivery	13 (87%)	14 (100%)
Controlled cord traction & uterine massage	13 (87%)	14 (100%)
Dry baby immediately after delivery	15 (100%)	14 (100%)
Place the baby on mother’s abdomen	13 (87%)	11 (79%)
Weigh baby after delivery	13 (87%)	14 (100%)
Initiate breast feeding within one hour	15 (100%)	14 (100%)
Basic emergency obstetric care, %		
Parenteral Magnesium sulphate/Diazepam for convulsions	11 (73%)	13 (93%)
Parenteral antibiotic	14 (93%)	14 (100%)
Parenteral oxytocin for haemorrhage	14 (93%)	14 (100%)
Manual removal of placenta/retained products	10 (67%)	12 (86%)
Delivery with vacuum extraction or forceps*	0 (0%)	8 (57%)
Induction of labour	6 (40%)	10 (71%)
Injection Dexamethasone/ Betamethasone to mother for premature labour	12 (80%)	9 (64%)
New born resuscitation with bag and mask	14 (93%)	14 (100%)
Injectable antibiotics for newborn sepsis	10 (67%)	10 (71%)
Comprehensive emergency obstetric care, %		
Caesarean section	0 (0%)	4 (29%)
Blood storage	0 (0%)	2 (14%)
I/v fluids for newborns	8 (53%)	10 (71%)
Oxygen for newborns	0 (0%)	4 (29%)
Deliveries conducted per centre over 6 months; median (IQR)	100 (60–131)	111 (64–293)
Referred during labour per centre; median (IQR)**	20 (19–25)	36 (23–43)

*facility is available but not practiced regularly; **data available from 10 CHCs and 9 PHCs only

We obtained ethics approval from the London School of Hygiene and Tropical Medicine and Indian Institute of Public Health-Hyderabad. (LSHTM Ethics Ref: 9613; IIPHH Ethics Ref: IIPHH/TRC/IEC/009/2014) All the eligible staff provided written consent to participate.

## Results

### Provision of obstetric care at the study centres

The provision of antenatal care, appropriate management of complications and onward referral depended on the designation of the staff (doctor, staff nurse, ANM), and the infrastructural support at the centres. Table 1 describes characteristics of the study participants. We interviewed 49 doctors and staff nurses from CHCs and 35 from PHCs, and 63 ANMs from SHCs across 34 CHCs and PHCs, and 40 SHCs. The staff from SHCs had an average 15 years of experience, while those at PHCs and CHCs, they had an average 10 years of experience. A large proportion of staff in PHCs (78%) had received the Skilled Birth Attendant (SBA) training, with less in CHCs (68%) and the lowest proportion in SHCs (50%). With respect to the designation, 87% of the staff nurses were SBA trained, while a lower proportion of doctors (58%) and ANMs (50%) were SBA trained. Two-fifth staff at PHCs and one-third from CHCs were trained to use the Safe Childbirth Checklist. All of these were from Andhra Pradesh. Himachal Pradesh had not introduced Safe Childbirth Checklist by the time of this study.

The obstetric head of the hospitals mentioned the services routinely provided at their centre in the past 3 months. Table 2 describes the childbirth services told available at PHCs and CHCs under study. The staff mentioned that referrals early in pregnancy depended on the childbirth facilities that could be provided at the centre later.

The SHCs were only responsible for early registration of pregnancy, preliminary history taking and examination for screening high-risk, basic antenatal care (Iron folic acid supplementation, tetanus toxoid immunization), and appropriate advice for healthy pregnancy and birth planning. SHCs from Himachal Pradesh were an exception, where ANMs assisted childbirth at home or the centre if the mother arrived very late in labour or delivered at home. Staff from Himachal Pradesh mentioned that at PHCs and CHCs, antenatal check-ups were done by ANM supervisor or nurses and if the antenatal woman required higher level of care or special investigations they were referred for a doctor’s consultation. In Andhra Pradesh, antenatal care at PHCs and CHCs was mostly conducted by a team of a doctor and a nurse. The PHCs and CHCs were BEmOCs with plus/minus few emergency obstetric signal functions. Three CHCs had caesarean section services and only had blood storage.

## Knowledge, practices and attitude on screening and referral for high-risk and early complications in the antenatal period

Staff enumerated several high-risk and early complications that should be screened for in antenatal women (Fig. 1a and c), and that should be referred on from their health centre during the antenatal period (Fig. 1b and d). These were unprompted responses.

### High-risk in pregnancy

Between half and three-quarters of the staff mentioned screening for history of caesarean section and history of abortion/stillbirth/preterm. Multiple foetus and abnormal lie of the foetus were less frequently mentioned. In general, a higher proportion of ANMs at SHCs enumerated the high-risk in pregnancy for screening compared to doctors and staff nurses at PHCs and CHCs (Fig. 1a). Most staff at SHCs, almost half the staff at PHCs, and less than a quarter of the staff at CHCs, mentioned referring antenatal women for any high-risk factor (Fig. 1b). Most commonly mentioned were previous caesarean section and previous abortions.

We further enquired individually for common high-risk conditions during pregnancy to assess the practices and attitudes of staff (Fig. 2). Most of the staff at all centres were confident to manage ANCs but not deliveries. More than half of staff at PHCs but only a quarter staff at CHCs would refer a woman for delivery if she had a previous caesarean section, multiple foetus or abnormal lie of foetus. A quarter of the staff at PHCs felt that ANC care for such women should not be provided at their centre. Staff suggested that the women with high-risk pregnancies should register at more advanced centres where they could plan for delivery, and get all the ANC there.

**Table 3:** Practice, problems and suggestions regarding referral during antenatal care, reported by health staff working at CHC/PHC/SHC

	SHC, *N* = 63	PHC, *N* = 49	Sub-district hospital /CHC, N = 35
Components of referral practice, %			
Prepare referral note	41	63	69
Counsel	65	57	74
Advise to call ‘108’ in case of emergency	75	69	74
Arrange transport	8	6	14
Communicate via phone	70	39	14
Provide stabilising care	13	18	14
Problems faced in referring antenatal women			
• Patients are uncooperative, they refuse to go to higher centres –PHC & CHC			
• Transport is not available in remote villages. ‘108’ ambulances are sometimes late –PHC in HP			
• No transport for antenatal elective or emergency referral –PHC in HP			
• ANM supervisor conducted ANC and referred by herself –PHC in HP			
• Nurse experienced and willing to provide care, but in-experienced doctor suggested referral –PHC in HP			
• Not a delivery point, so all pregnant women referred to the delivery point –PHC in HP			
• Not clear about when to refer. Mostly refer when doctor is not available –PHC & CHC			
• Refer to District hospitals on weekend, as doctors may not be available at CHC/Sub-district hospital-PHC in HP			
• Referral not accepted at higher centre –PHC in HP			
• ANC referrals usually from the outpatient clinic and there was no record maintenance –PHC & CHC			
• Lab technician not available to provide basic investigations –PHC & CHC in HP			
• No information on any change in services and availability of blood at the higher centre –PHC			
Support required from system to improve referrals for antenatal women			
• Transport facility for emergency antenatal care cases –PHC & CHC			
• Need access to obstetrician. In case of any high-risk or complication, the patient needs to visit an obstetrician at least once –PHC & CHC			
• Call centre support to discuss difficult cases			
• Training required to upgrade knowledge and skills for high-risk and complication cases			
• Support from senior staff and doctor to allow ANC care and help in decision making for management of difficult cases			
• Need more staff. PHCs should have two medical officers and at least 3–4 staff nurses and one lab technician –PHCs in HP			
• Moral support from the system and senior staff should support our decisions			
• Lab technician required at seven PHCs and 2 Sub-district hospitals; radiology services required for USG at CHC or Sub-district hospital.			
• Blood bank and better testing facilities for thyroid and diabetes so that more women can be managed at CHC/Sub-district hospitals			

### Early complications in pregnancy

Regarding knowledge, almost all the staff knew about the need to screen for high blood pressure and anaemia in antenatal women. Between half and three-quarters of the staff mentioned eclampsia/convulsions, bleeding in pregnancy, diabetes/thyroid disease, swelling of feet, foetal distress and HIV status as conditions to be screened for. The distribution was similar across the SHCs, PHCs, and CHCs (Fig. 1c).

Figure-1e shows the list of investigations that were suggested for antenatal women at the respective health centres. These were unprompted responses. Almost all staff listed blood haemoglobin levels, and approximately 90% enumerated blood grouping, urine albumin, random blood sugar, HIV and HbsAg tests as part of antenatal work-up. Ultrasonography (USG) of the abdomen was listed by about three-quarters and VDRL (test for syphilis) by half of the staff. These proportions were slightly lower in ANMs at SHCs. Almost all the staff enumerated referral for antenatal women with severe anaemia and high blood pressure. Foetal distress was the least mentioned condition. Other early complications were mentioned only by a quarter to half of the staff. Staff from all three types of centres mentioned similar referral indications (Fig. 1d). Compared to Andhra Pradesh, a higher proportion of ANMs from Himachal Pradesh mentioned various high-risk conditions and early complications to be screened and referred (data not shown).

Regarding attitudes and practice, more than three quarters staff were confident in providing antenatal care in cases of swelling of feet, high fever and high blood pressure, moderate to severe anaemia and foetal distress (Fig. 2). However, they lacked confidence in managing delivery in such cases, this was lower among staff from PHCs and lowest among staff from SHCs). Only half staff at CHCs and a quarter at PHCs were confident they could manage antenatal care for women with eclampsia, bleeding in pregnancy, and HIV positivity while another quarter of staff at PHCs mentioned that these conditions should not be managed at their centres. A very small proportion was confident to manage cases of diabetes or thyroid disease, most also felt that these should not be managed at their centres.

We observed that the the level of understanding of the aspects in antenatal management varied across centres. ANMs at SHCs considered their role in management to include screening for high-risk and early complication, referral when appropriate, and continuing to provide basic antenatal care throughout pregnancy. Staff nurses and doctors at PHC and CHCs understood that providing treatment for early complications or stabilising care before a referral was also part of management.

Overall, 47 of 147 (31%) mentioned screening for at least 10 of the 16 common high-risk conditions and early complications of pregnancy. Only 35% (17 of 49) of the staff at Primary health centres, and 51% (18 of 35) at Community health centres, mentioned that they managed at least 10 listed conditions and, the remaining staff referred most of such cases early in pregnancy.

A few staff from SHCs and PHCs asked for training to manage women with swelling in their feet, high blood pressure, and anaemia. Roughly a quarter staff at CHCs expressed a need for training or extra resources to be able to manage women with high blood pressure, eclampsia and bleeding in pregnancy.

### Pre-referral management of specific antenatal case scenarios

#### Moderate anaemia in the 2nd trimester (figure-3a)

All the staff across all the centres diagnosed moderate anaemia correctly and a half would refer such women, whereas per guidelines PHCs and CHCs should be able to manage all moderate anaemia cases. More than 80% of staff prescribed oral iron and Folic-acid tablets (100 mg) twice in a day. Only one-third in PHCs, and almost half the staff in CHCs, prescribed injectable iron for management. Injectable iron was mentioned less often by the staff from Himachal Pradesh. Although Mebendazole tablets and nutrition advice are recommended in India’s RCH programme, a negligible number of staff mentioned these.

#### Pregnancy-induced hypertension in the 3rd trimester (Fig.-3b)

All the staff made a correct diagnosis. The 94% from SHCs, 85% at PHCs and 45% staff at CHCs, referred the women for higher care. Forty-two percent from PHCs and 71% from CHCs prescribed anti-hypertensive drugs available at their centres. Blood pressure monitoring was mentioned by 50% staff at CHCs and a lower proportion at PHCs and SHCs. Magnesium sulfate prophylaxis, rest and low salt diet were less frequently mentioned.

#### Labour pains at 30 weeks (Fig. 3c)

Almost half at PHCs and two-thirds staff at CHCs would assist delivery of a preterm birth. About two-thirds also mentioned that they would refer the women to an advanced centre if the cervix was not fully dilated. Only a few staff from PHCs and SHCs mentioned injection Dexamethasone and none mentioned tocolytics. Two-fifths of staff at CHCs would give a prophylaxis injection of Dexamethasone if labour could be delayed, but only three doctors suggested tocolytics to delay labour. On discussion, it appeared that they were unaware that tocolytics were a line of management provided at some higher centres.

**Fig. 3:**
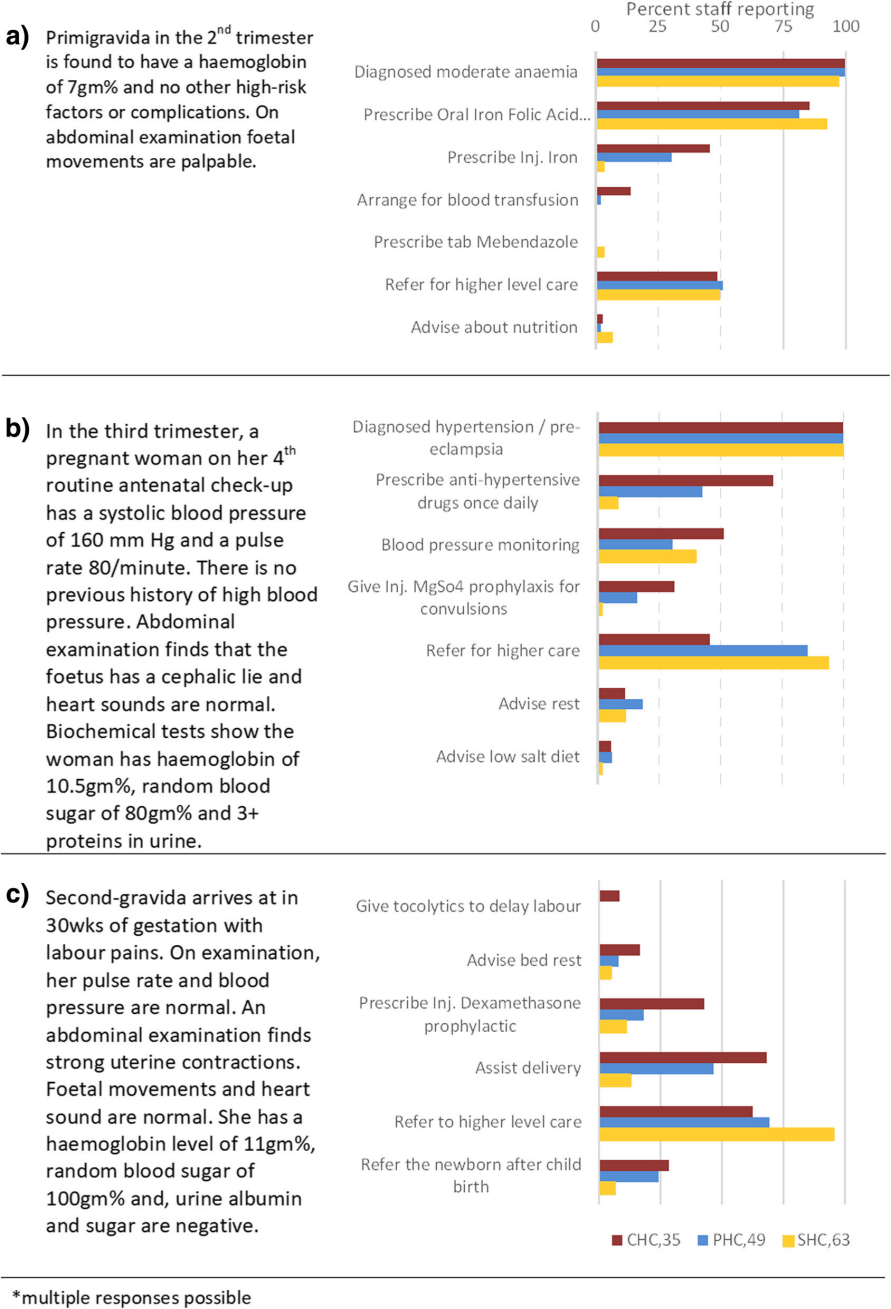
Management of common high-risk or early complication in pregnancy*. (Legend shows the N for each type of facility)

### Appropriate referral in antenatal period- referral Centre, communication, and transport

Table-3 describes the administrative steps taken while referring, and the problems faced and requested support by the providers to help them improve quality of antenatal care and referral management.

A large proportion of ANMs at SHCs said they informed the ANM supervisor or staff nurse at the referral centre (PHC or CHC) by telephone. This proportion was lower in PHCs (39%) and lowest in CHCs (14%). We rarely found a referral note being provided. There were no written records on referrals during ante-natal care in facility registers. Almost all referrals were from out-patient clinics and only three PHCs and three CHCs had some mention of referrals in the out-patient clinic register.

Decisions about which referral centre to choose depended on the indication for the referral and the facilities available at the higher public health centre. Only two staff acknowledged that they referred patients to private facilities. ANMs at SHCs mentioned that they referred women to Sub-district and District hospitals only in cases of acute emergency and on Sundays. For other routine check-ups, they said they referred the women to the nearest health centre (PHC or CHC). Some of the referrals were done for basic investigations because lab-technicians were not available at the PHC or CHC. The referred women had to re-fill the forms and re-consult the doctor at the referral centre. They often suffered more than the women reaching the referral centre directly. A few centres that did not have childbirth services (SHCs) referred women to register at higher centres (double registration) to make it easier for them to get admission there at the time of delivery. PHCs usually had only one doctor who on many occasions was unavailable to provide emergency care. Thus, referrals were higher in doctor’s absence, even for small ailments. The staff stated that most patients were unwilling to go to higher centres, and that several tribal and interior rural areas did not have transportation. In other places, free ‘108’ or‘102’ transport was available for only emergencies. Staff mentioned that on several occasions the referred women did not receive the expected care at the higher centre, thus indirectly questioning the value of referral. The services at higher centres were not standardised and changed with respect to the availability of resources including staff. There were no means of updating this information at a lower level.

Staff nurses from three PHCs demanded support from the doctors to allow them to provide better antenatal care and support in decision making to provide emergency treatment. Staff felt that pregnant women should have access to an obstetrician at least once. This could also help staff to manage the women at high-risk and with early complications at their centre as per the advice of the obstetrician. Staff requested vacant staff positions be filled and lab facilities be upgraded to be able to test for gestational diabetes and thyroid disease at PHCs and CHCs. Doctors also suggested a call centre facility to discuss difficult cases and support decision making. Several staff nurses and ANMs requested training to upgrade their knowledge and skills. Almost all doctors would value more moral support from senior officials, and requested that the system should respect and stand by their clinical decisions in difficult situations, especially in times of conflict with the community.

## Discussion

Our study was able to assess the quality of obstetric and referral care across a range of peripheral health care centres, and types of personnel, in the study districts. The survey data enabled a thorough analysis of obstetric practice and referral to higher levels of care for women with obstetric high-risk and complications. The survey data were supplemented by information from facility checklists, and formal and informal interviews, to enable a comprehensive analysis.

We found that routine antenatal care and screening was largely provided by ANMs in SHCs, and staff nurses at PHCs and CHCs. A higher proportion of ANMs at SHCs knew about screening for high-risk conditions and early complications, compared to nurses and doctors, but that the ability to manage complications during pregnancy was poor and ANMs did not provide any first-aid before referral. A study from northern Karnataka, India also found that ANMs were more confident than staff nurses to manage routine antenatal care and to identify complications [24].

Regarding screening, high-risk conditions and early complications in pregnancy that involved taking a case history and lab tests were more likely to be mentioned by health staff than conditions screened by general examination (except blood pressure) and abdominal examination. This may be due to time constraints for examinations because of high patient load, or due to over-reliance on lab investigations. A study from Pakistan reported the reverse, where history taking was observed in less than 30% pregnant women while examination was for 50% or more [25]. Although the health centres in our study did not have the capacity to conduct all recommended lab tests, the staff mostly referred pregnant women for such tests. The proportion of respective tests advised in our study, were similar to findings from Belgaum and Nagpur in India [17]. Studies from other LMIC reported that between 30 and 70% of women received the basic recommended lab investigations at peripheral health centres in the antenatal period [25–29].

In all, only a third of staff in our study mentioned screening for at least 10 of the 16 mentioned common high-risk conditions and early complications in pregnancy. In a study from Malaysia, 35% antenatal women were assessed according to the complete risk assessment criteria [30]. Other studies from LMIC have also found that only a quarter to a half of the antenatal women received a good or moderate quality of antenatal care [18, 19, 25, 27, 28, 31–33]. Low adherence to minimum levels of recommended ANC content was also observed in high-income country settings [34–36]. Due to missed opportunities for screening and early management, women may present with the advanced disease later and receive a delayed referral. Studies also found that screening had greater adherence than health education or other prescriptions [19, 25, 31, 36].

As per guidelines, moderate anaemia should be easily managed at CHCs and PHCs [37] but a considerable proportion of staff in our study mentioned that they would refer the case to higher centres. Knowledge of diagnosis and management of pre-eclampsia was good among nurses and ANMs, but most staff did not administer MgSO4 or antihypertensive treatment to women with pre-eclampsia. Our results are very similar to a study from six African countries where provider knowledge about diagnosis of pre-eclampsia/ eclampsia exceeded 80% however, knowledge of first actions to be taken varied from 33 to 77%, and action to be taken in the event of a convulsion did not exceed 51% in any country [38]. Nurses were mostly unaware of dosage and route of administration [24]. Only one-third of care providers prescribed an injection of Dexamethasone or Betamethasone for preterm labour in our study, this was observed to be nil in the peripheral health centres in a study from 6 LMIC countries. At higher centres, corticosteroids were administered between 2 and 12% in five LMIC and 44% in Argentina [39].

Most studies on the quality of antenatal care from LMIC have assessed practice through case records and observations. Our study comprehensively assessed providers’ perspective on the provision of antenatal care. Most staff felt that it was better to refer women presenting with an existing high-risk condition or complication in early pregnancy. The staff were either under-confident did not have enough resources or felt that the complications should be managed only by obstetricians. The staff feared blame should the complication worsen and lead to maternal death. Another few mentioned that they were discouraged by the district administration to manage complications at their centre. Examples of these conditions were pre-eclampsia, gestational diabetes and previous caesarean section, which should be well managed at CHCs. CHCs in India are meant to operate as a CEmOC or at least a BEmOC level, that can manage common obstetric complications [37], but this was not the case in our study.

Overall, a large proportion of antenatal referrals were likely to be very early, unnecessary, or for investigations, and a smaller proportion were delayed referrals. This, coupled with poor pre-referral management, will worsen pregnancy outcomes. Inappropriate referrals may make pregnant women choose a private facility closer to home or a tertiary hospital which could provide the necessary care throughout pregnancy and childbirth. Counselling for birth planning for such high-risk conditions and complications was not seen. Reluctance to manage at the appropriate level can lead to over reliance on tertiary facilities [40–42]. This in turn causes over-crowding at tertiary facilities, consequently leading to negligent and poor quality care, over reliance on augmentation, inappropriately short lengths of stay, poor infection prevention and hospital acquired infections, women lying on floors, commodity shortages, and greater expense.

A systematic review from India found that a high proportion, between 25 and 52%, of all antenatal women were referred due to a high-risk condition or complication [41]. This is due to the inability of primary health centres to provide basic emergency antenatal and delivery care, and a tendency for unjustified referrals to higher care institutions [41]. We observed high antenatal referrals but none of the interventions to improve transport for pregnant women in India provide transportation for elective antenatal referrals. Analysis of ‘108’ ambulance data found that less than 1% of all the pregnant users of the ‘108’ services used it for antenatal care or complications such as abortions. This proportion is far less than the estimated total burden of antenatal referrals in the population (25–52%) [41]. In such cases, extremely poor women may not travel to higher level centres if they do not perceive the importance of a referral. However, in the case of a complication that requires immediate transfer, the ‘108’ and ‘102’ ambulance service, as well as Janani Express Yojana, would transport such women [43]. Currently, state governments are willing to use government run ‘102’ service for elective antenatal referrals for high-risk and complication cases [43]. Currently, there are no records and no established communication channels for antenatal referrals.

Our study also had a few limitations. The practices of health staff were assessed by interviewing them rather than direct observations. The staff may have over-stated or under-stated actual practice, but we found that our findings were consistent with findings from other observational studies from India. We also noticed that findings related to knowledge, practice, and attitude were consistent with each other, and with those from the open ended questions. We did not have a planned explicit qualitative in-depth enquiry in this study, but interviews spontaneously extended into a discussion of other systemic issues contributing to the quality of antenatal care. We suspect social desirability bias and possibility of shifting the blame to others or the system.

Referral systems are peculiar to the health systems they are embedded in. Thus they should be developed and adapted to the local needs [44]. The results of our study suggest that to improve obstetric outcomes in India, emphasis must be placed on health systems strengthening (including human and material resources, protocols and services), with added focus on decisions for referral and quality of pre-referral stabilising care. Abridged protocols and referral guidelines for each level of health facility, coupled with continuous mentoring will empower the staff in peripheral facilities, and will also provide the opportunity to monitor the practices against standards for that level of care. Other recommendations regarding referrals are that mechanisms should be devised to ensure the availability of transport, adherence to referral advice, and to improve communications across levels of care [44]. The health systems should respect the needs and concerns of providers, provide feedback and moral support. It is recommended that administrations should strengthen documentation for case sheets, registers and reports, especially at lower centres [42, 45]. Transport services for elective antenatal referrals along with emergencies should be improved for the poor and remote regions. Finally, the Reproductive Child and Health programme should include process and outcome indicators for assessing quality of obstetric care, and appropriate referral and transfers [45].

## Conclusion

Staff in peripheral public health centres had sub-optimal knowledge of, and practices for, screening of common high-risk conditions and complications in pregnancy. There were large gaps in knowledge of emergency care for obstetric complications. Knowledge of antenatal screening among ANMs at SHCs was better than the staff at PHCs and CHCs, but management by ANMs was poorer. CHCs were supposed to provide BEmOC but only a quarter to a half of staff managed common antenatal complications. A large proportion of staff from PHCs and CHCs referred pregnant women with high-risk conditions or early complications in pregnancy after giving some treatment. ANMs referred most early complications without providing any treatment. Some referrals were for routine lab investigations and ultrasonography. Staff generally lacked confidence, or did not have resources, or felt that some complications should only be managed at higher levels by obstetricians. Staff desired skill building, mentoring, and moral support and motivation from senior officers.

We conclude that the health systems should improve the provision of obstetric care in India by standardising services at each level of health care, and increase the focus on immediate care for complications, appropriate decision-making for referral, and improving referral communication. Indicators to monitor referrals should be incorporated into plans for monitoring quality of obstetric care.

## Data Availability

The data are not publically available, however, can be made available upon reasonable request to the corresponding author.

## Abbreviations

ANM: Auxillary Nurse Midwife
BEmOC: Basic Emergency Obstetric Care
CEmOC: Comprehensive Emergency Obstetric Care
CHC: Community Health Centres
LMIC: Low Middle Income Country
MgSO4: Magnesium Sulphate
PHC: Primary Health Centres
SBA: Skilled Birth Attendant
USG: Ultra-sonography
WHO: World Health Organisation
